# Stimulation of Peritoneal Mesothelial Cells to Secrete Matrix Metalloproteinase-9 (MMP-9) by TNF-α: A Role in the Invasion of Gastric Carcinoma Cells

**DOI:** 10.3390/ijms19123961

**Published:** 2018-12-09

**Authors:** Teruaki Oku, Kentaro Shimada, Hiroki Kenmotsu, Yusuke Ando, Chisato Kurisaka, Rikio Sano, Makoto Tsuiji, Shinya Hasegawa, Tetsuya Fukui, Tsutomu Tsuji

**Affiliations:** 1 Department of Microbiology, Hoshi University School of Pharmacy and Pharmaceutical Sciences, Tokyo 142-8501, Japan; oku@hoshi.ac.jp (T.O.); m842@hoshi.ac.jp (K.S.); s061084@hoshi.ac.jp (H.K.); p17andoh@hoshi.ac.jp (Y.A.); d1403@hoshi.ac.jp (C.K.); m220@hoshi.ac.jp (R.S.); m-tsuiji@hoshi.ac.jp (M.T.); 2 Department of Health Chemistry, Hoshi University School of Pharmacy and Pharmaceutical Sciences, Tokyo 142-8501, Japan; s-hasegawa@hoshi.ac.jp (S.H.); fukui@hoshi.ac.jp (T.F.)

**Keywords:** TNF-α, matrix metalloproteinase, peritoneal mesothelial cell, gastric cancer, metastatic dissemination

## Abstract

It has recently been recognized that inflammatory cytokines, such as tumor necrosis factor-α (TNF-α), upregulate the secretion of matrix metalloproteinase-9 (MMP-9) from cancer cells and thereby promote peritoneal dissemination. In this study, we found that TNF-α also stimulated peritoneal mesothelial cells to secrete MMP-9 as assessed by zymography. MMP-9 gene expression in mesothelial cells induced by TNF-α was confirmed by quantitative RT-PCR analysis. We then utilized the reconstituted artificial mesothelium, which was composed of a monolayer of mesothelial cells cultured on a Matrigel layer in a Boyden chamber system, to examine the effects of TNF-α on carcinoma cell invasion. The transmigration of MKN1 human gastric carcinoma cells through the reconstituted mesothelium was promoted by TNF-α in a dose-dependent manner. The increased MKN1 cell migration was partially inhibited by the anti-α3 integrin antibody, indicating that the invasion process involves an integrin-dependent mechanism. Finally, we observed that the invasion of MMP-9-knockdown MKN1 cells into Matrigel membranes was potentiated by the exogenous addition of purified proMMP-9. These results suggest that TNF-α-induced MMP-9 secretion from mesothelial cells plays an important role in the metastatic dissemination of gastric cancer.

## 1. Introduction

Metastatic peritoneal dissemination causes a poor prognosis for patients with advanced gastric cancer. Cancer metastasis depends on multiple interactions between cancer cells and various factors derived from the host microenvironment, including cytokines, motility factors, cell adhesion molecules, and matrix-degrading enzymes. On the other hand, many recent studies have suggested the relevance of inflammation to cancer progression. In particular, tumor necrosis factor-α (TNF-α) and other inflammatory cytokines have been shown to participate in the initiation and progression of cancer [1,2,3,4,5]. TNF-α is a multifunctional cytokine that modulates various aspects of cancer cell phenotypes, such as cell proliferation, migration, invasion, and metastatic potential, in addition to causing the death of cancer cells. TNF-α, either alone or in combination with transforming growth factor-β, has also been shown to induce so-called epithelial-mesenchymal transition (EMT), which includes changes in both morphological and invasive phenotypes [6,7]. It has been well established that TNF-α induces the production of matrix metalloproteinases (MMPs) by carcinoma cells originating from various tissues, and these MMPs facilitate cancer cell invasion and metastasis [8,9,10,11,12,13]. A recent clinical study of patients with gastric cancer indicated that TNF-α expression was significantly different between patients with and those without peritoneal metastasis and was one of the risk factors for peritoneal metastasis of gastric cancer [14].

We have studied the interaction of gastric carcinoma cells with the extracellular matrix (ECM) deposited by peritoneal mesothelial cells and found that MKN1 human gastric carcinoma cells secreted MMP-9 accompanied by enhanced invasion through Matrigel-reconstituted basement membranes when these cells adhered to ECM containing laminin-332, a major component of submesothelial basement membranes [15]. Moreover, the phenotype of MKN1 cells was found to be more invasive in the presence of TNF-α derived from tumor-associated macrophage-like cells in association with enhanced MMP-9 secretion [16]. In the present study, we co-cultured MKN1 cells with mesothelial cells isolated from murine peritoneum and examined the effects of treatment with TNF-α on MMP-9 production by these cells. The TNF-α treatment of these cells resulted in the induction of MMP-9 secretion not only from MKN1 carcinoma cells but also from mesothelial cells. This result prompted us to characterize the enhanced MMP-9 production by mesothelial cells upon TNF-α treatment. We also present evidence showing that MMP-9 secreted from mesothelial cells plays a significant role in the invasion of gastric carcinoma cells. For this purpose, we used a reconstituted mesothelium consisting of peritoneal mesothelial cells and Matrigel basement membranes in a Boyden chamber system.

## 2. Results

### 2.1. Mesothelial Cells Secreted MMP-9 in Response to Stimulation with TNF-α

**Figure 1:**
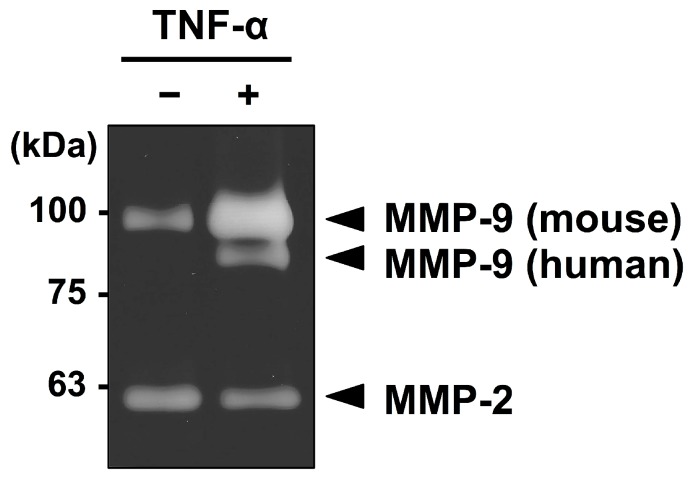
Zymographic analysis of MMPs in the conditioned medium of the co-culture of MKN1 and mesothelial cells after TNF-α treatment. A suspension of MKN1 cells (5 × 10^4^ cells/0.2 mL) in ASF104 medium was added to a monolayer of murine peritoneal mesothelial cells in a 96-well culture plate and the cells were cultured with/without TNF-α (10 ng/mL) for 48 h. The culture supernatant was analyzed by gelatin zymography.

**Figure 2:**
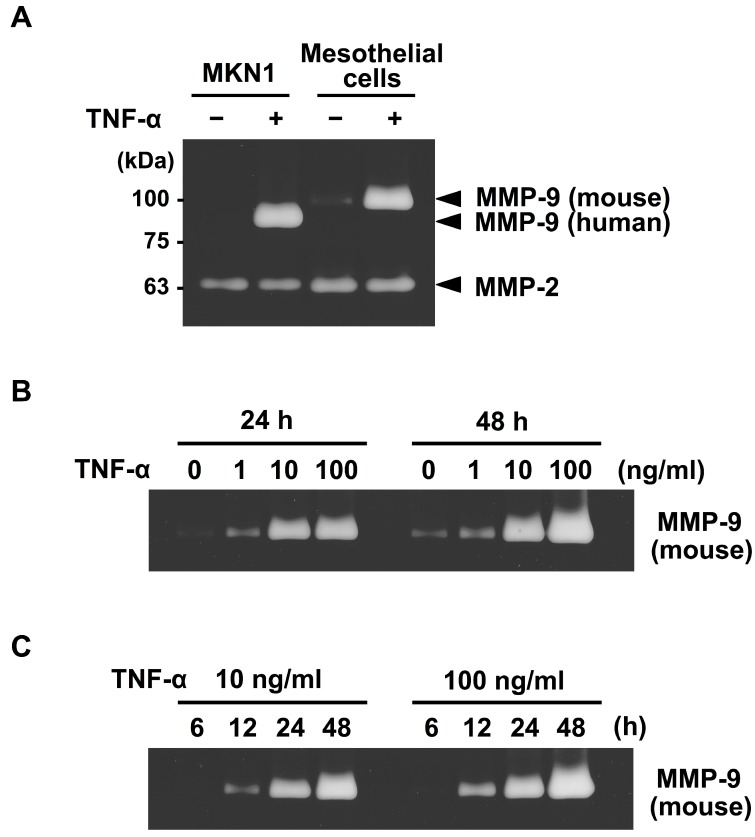
Effects of TNF-α on the secretion of MMP-9 from peritoneal mesothelial cells as assessed by zymography. (**A**) Monolayers of peritoneal mesothelial cells and MKN1 cells were separately cultured in ASF104 serum-free medium with/without TNF-α (10 ng/mL) for 48 h. (**B**) Mesothelial cells were cultured with TNF-α (0, 1, 10 or 100 ng/mL) for 24 or 48 h. (**C**) Mesothelial cells were cultured with TNF-α (10 or 100 ng/mL) for 6, 12, 24 or 48 h.

We first examined the effects of TNF-α on MMP-9 secretion in the co-cultures of MKN1 and peritoneal mesothelial cells. Zymographic analysis of the conditioned medium of the co-culture of these cells revealed that MMP-9 secretions from both MKN1 and murine peritoneal mesothelial cells were markedly increased in the presence of TNF-α (10 ng/mL) (Figure 1). Since murine MMP-9 has a slightly higher molecular mass (105 kDa for proMMP-9) than its human counterpart (92 kDa) due to the presence of an extra amino acid sequence in murine MMP-9 [17], the two molecules can be distinguished from each other by zymography. By contrast, the MMP-2 level was not significantly changed in the conditioned medium after TNF-α treatment. The upregulation of MMP-9 secretion from both types of cells by TNF-α treatment (10 ng/mL) was confirmed when TNF-α was added to the separate cultures of MKN1 and mesothelial cells (Figure 2A). We observed low-level secretion of MMP-2 from both cell types in the absence of TNF-α, and the secretion was not substantially influenced by TNF-α treatment in either case. We then examined the dose-response of the potentiation of MMP-9 secretion by TNF-α. In the zymographic analysis, we detected MMP-9 activity in the conditioned medium of mesothelial cell culture in the presence of TNF-α (1 ng/mL) for 24 or 48 h, and the MMP-9 secretion was dose-dependently increased (up to 100 ng/mL) (Figure 2B). In the kinetic analysis, we detected MMP-9 activity after the culture with TNF-α for 12 h, and MMP-9 secretion was increased at 24 and 48 h (Figure 2C). These results clearly indicated that TNF-α treatment induced MMP-9 secretion from mesothelial cells, in addition to enhancing MMP-9 secretion from MKN1 gastric carcinoma cells.

**Figure 3:**
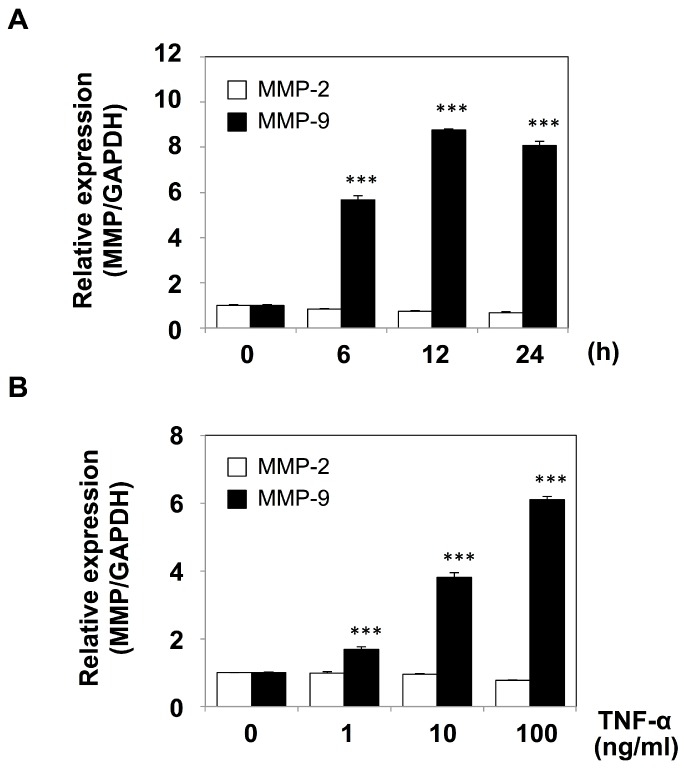
RT-qPCR analysis of mRNA for MMP-2 and MMP-9 in mesothelial cells after treatment with TNF-α. (**A**) Mesothelial cells were cultured in ASF104 serum-free medium containing TNF-α (10 ng/mL) at 37 °C for 0, 6, 12 or 24 h. Total RNA was then isolated, and RT-qPCR for the genes for MMP-2 (*open bar*) and MMP-9 (*closed bar*) was carried out as described in Section 4. (**B**) Mesothelial cells were cultured in ASF104 medium containing TNF-α (0, 1, 10 or 100 ng/mL) for 6 h. The gene expressions of MMP-2 (*open bar*) and MMP-9 (*closed bar*) were analyzed by the relative standard curve method using Gapdh as an internal control. Experiments were performed in triplicate, and the data are presented as the mean ± SEM. Statistical data analysis was conducted using the Student’s *t*-test. *** *p* < 0.005 vs. control.

We next conducted RT-qPCR to measure mRNA levels for MMP-9 in mesothelial cells after TNF-α treatment. The results shown in Figure 3A indicated that the mRNA level in mesothelial cells was elevated at 6 h after the stimulation by TNF-α (10 ng/mL) and reached the maximum level at 12 h, where it remained until 24 h after the stimulation. The increase in MMP-9 mRNA expression was found to be dose-dependent within the range of 1–100 ng/mL of TNF-α (Figure 3B), which was in good agreement with the results of the zymographic analysis (Figure 2B). However, the mRNA for MMP-2 exhibited no significant change after TNF-α treatment.

### 2.2. TNF-α Potentiates MKN1 Cell Invasion through the Reconstituted Mesothelium

**Figure 4:**
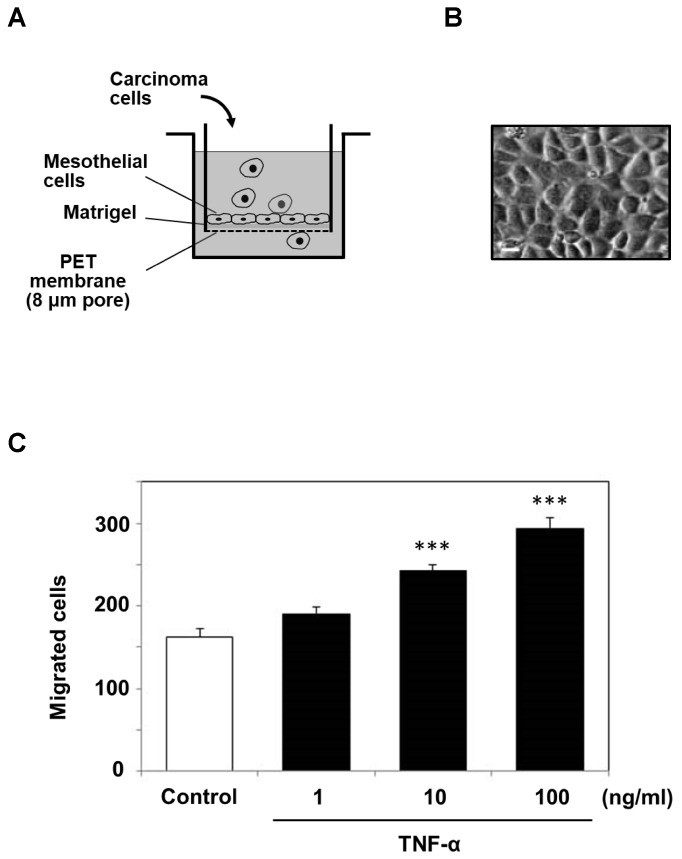
Cell invasion assay using a reconstituted artificial mesothelium in a Boyden chamber (Transwell) system. (**A**) The inner chamber with a membrane (8.0 μm pore) was composed of a monolayer of peritoneal mesothelial cells on a Matrigel layer and was utilized to examine the migration of MKN1 cells. The outer chamber was filled with ASF104 medium supplemented with HT1080 serum-free conditioned medium as a chemoattractant. (**B**) Microscopic observation of a monolayer of mesothelial cells (scale bar = 20 μm). (**C**) After mesothelial cells were treated with TNF-α (1, 10 or 100 ng/mL) and washed with ASF104 medium, MKN1 cells (1 × 10^5^ cells/0.2 mL) were placed in the inner chamber and incubated at 37 °C for 16 h. The cells migrating into the outer chamber through the membrane were counted under a microscope after staining with Diff-Quik. Experiments were performed in triplicate, and the data are presented as the mean ± SEM. Statistical data analysis was conducted using the Student’s *t*-test. *** *p* < 0.005 vs. the control.

Because the above experiments indicated that mesothelial cells secreted MMP-9 in response to TNF-α treatment, we designed an artificial, reconstituted mesothelium where a monolayer of mesothelial cells was cultured on a Matrigel layer in a Boyden chamber system (Figure 4A) and examined the effects of TNF-α on carcinoma cell invasion. Mesothelial cells isolated from the murine peritoneum grew as a monolayer with polygonal morphology after 4–5 days (Figure 4B). The transmigration of MKN1 cells through the reconstituted mesothelium was promoted by TNF-α in a dose-dependent manner (Figure 4C).

**Figure 5:**
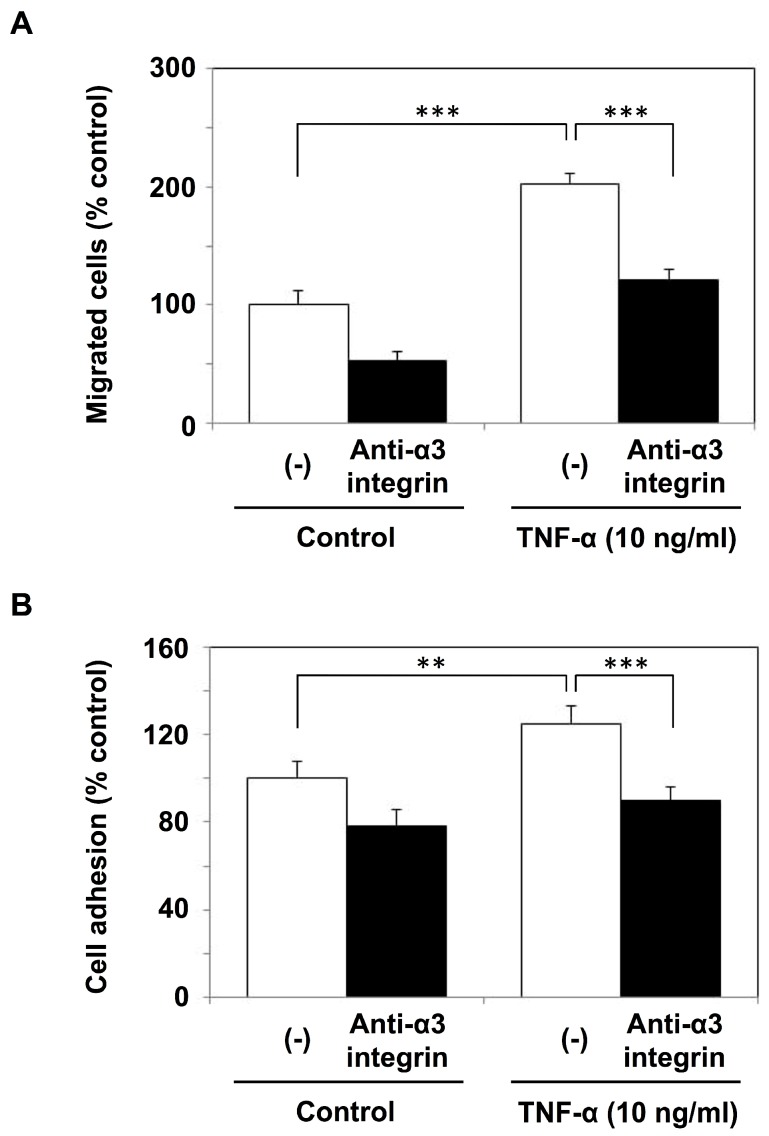
Invasion and adhesion of MKN1 cells and effects of the anti-α3 integrin antibody. A monolayer of mesothelial cells was stimulated with TNF-α (10 ng/mL) for 6 h. (**A**) MKN1 cells (1 × 10^5^ cells/0.2 mL) in ASF104 serum-free medium were added to the inner chamber of the reconstituted mesothelium and incubated at 37 °C for 16 h. The cells that had migrated into the outer chamber through the membrane were counted under a microscope. (**B**) Fluorescently labeled MKN1 cells were added to the monolayer of mesothelial cells in a 96-well culture plate, and incubated at 37 °C for 40 min. After non-adherent cells were removed by washing, adherent cells were lysed with 1% Nonidet P-40 and measured with a fluorescence spectrophotometer (Ex = 490 nm, Em = 520 nm). For the inhibition experiments, MKN1 cells were treated with the anti-α3 integrin antibody (SM-T1, 10 μg/mL) at 0 °C for 30 min. Experiments were performed in triplicate, and the data are presented as the mean ± SEM. Statistical data analysis was conducted using the Student’s *t*-test. ** *p* < 0.01, *** *p* < 0.005.

We previously found that the interaction between α3β1 integrin on cancer cells and laminin in the mesothelium played an important role in the cancer cell adhesion and invasion [15,18]. Next, we examined the effects of the anti-α3 integrin antibody on the transmigration of MKN1 cells through the reconstituted mesothelium. The cell invasion potentiated by TNF-α was significantly inhibited by the anti-α3 integrin antibody (Figure 5A), suggesting the importance of an α3β1 integrin-dependent process in the invasion. The adhesion of MKN1 cells to a monolayer of mesothelial cells was also increased after the TNF-α treatment of mesothelial cells and was partially inhibited by the anti-α3 integrin antibody (Figure 5B). Mochizuki et al. [19] reported that the treatment of mesothelial cells with TNF-α induced their morphological change followed by an increase in the areas of intercellular gaps. This process may cause exposure of the submesothelial extracellular matrix (ECM) in the intercellular gaps. Because laminin-332, a counter-ligand for α3β1 integrin, is a major component of submesothelial ECM, TNF-α treatment might facilitate the adhesion of MKN1 cells to the mesothelium via α3β1 integrin/laminin-332 interaction. In RT-qPCR analysis, we observed a slight increase in expression of the γ2 subunit of laminin-332 after TNF-α treatment of mesothelial cells (Figure S1), and this might also have caused the increased adhesion of MKN1 cells. 

### 2.3. The Exogenous Addition of MMP-9 Promotes MKN1 Cell Invasion

**Figure 6:**
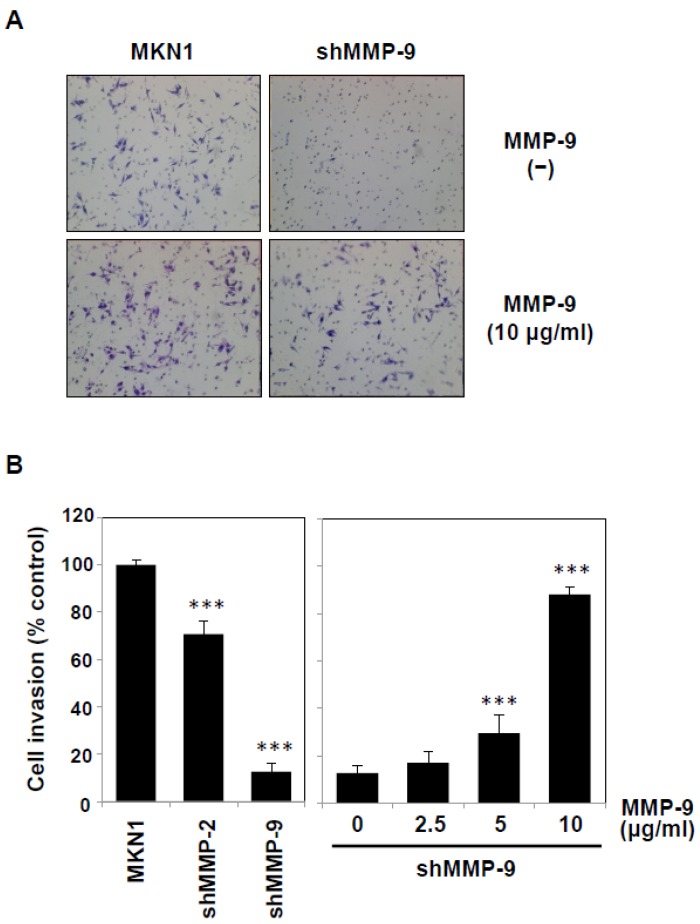
Potentiation of the invasion of MMP-9-knockdown MKN1 cells by exogenous MMP-9. MMP-2- or MMP-9-knockdown MKN1 cells were prepared by RNA interference as described in the Materials and Methods section. (**A**) The MMP-9-knockdown MKN1 cells (*right*) and parent cells (*left*) were assayed for in vitro invasion using Matrigel basement membranes in the presence or absence of MMP-9 purified from THP-1 cells. After culturing for 16 h, the cells that had migrated into the lower chamber were microscopically observed (40×). (**B**) MMP-2- or MMP-9-knockdown MKN1 cells were subjected to an invasion assay, and the migrated cells were counted under a microscope (*Left*). The invasion assay was performed in the presence of purified MMP-9 (2.5–10 μg/mL) (*right*). Experiments were performed in triplicate, and the data are presented as the mean ± SEM. Statistical data analysis was conducted using the Student’s *t*-test. *** *p* < 0.005.

Because our experiments demonstrated that TNF-α potentiated MMP-9 secretion from both mesothelial cells and cancer cells, we next addressed the effect of the exogenous addition of MMP-9 on the invasive behavior of cancer cells. When MMP-9 purified from human THP-1 leukemic cells (Figure S2) was added to the culture of MKN1 cells, their invasion into Matrigel basement membranes was increased by approximately 29% (Figure 6A). To exclude the contribution of secretion of MMP-9 and MMP-2 from cancer cells, we established MMP-knockdown cells by the RNA interference procedure (Figure S3). The silencing of MMP-9 expression in MKN1 cells resulted in marked impairment of the invasion (~90% inhibition), whereas MMP-2 silencing exhibited only a moderate effect (~30% inhibition) (Figure 6A,B). The addition of MMP-9 to the culture potentiated the invasion of MMP-9-knockdown cells in a dose-dependent manner (Figure 6B). These results suggest that the invasion of cancer cells is influenced by MMP-9 secreted from TNF-α-stimulated mesothelial cells.

## 3. Discussion

It has recently been recognized that inflammatory cytokines such as TNF-α play crucial roles in metastatic dissemination. For example, TNF-α treatment of human gastric cancer cells was shown to increase MMP-9 mRNA and protein expression levels [20]. In the present study, we demonstrated that peritoneal mesothelial cells also secreted MMP-9 in response to TNF-α (Figure 1 and Figure 2) and that the enhanced secretion of MMP-9 was associated with upregulation of MMP-9 transcription (Figure 3). In addition, we suggested the importance of the MMP-9 secreted by host mesothelial cells in gastric carcinoma invasion by utilizing a reconstituted mesothelium consisting of a monolayer of peritoneal mesothelial cells layered on a Matrigel basement membrane (Figure 4). The increase in cell invasion was shown to partly involve an integrin-dependent process (Figure 5). In addition to matrix-degrading enzymes secreted by the cancer cells themselves, MMP-9 derived from the host microenvironments, including peritoneal mesothelial cells, appeared to contribute to the cancer cell invasion into submesothelial matrices (Figure 6). Although mesothelial cells were thought to play a structural role in maintaining the smooth surface of the peritoneum and possess rather static properties, recent studies have shown that mesothelial cells produce various growth factors (e.g., vascular endothelial growth factor, basic fibroblast growth factor, epidermal growth factor, hepatocyte growth factor, and transforming growth factor-β) and ECM components (e.g., cytokeratin, vimentin and type I and III collagen) [21]. Therefore, peritoneal mesothelial cells are considered to be potentially active cells that dynamically respond to environmental changes. Chen and co-workers reported that TNF-α induced MMP-9 synthesis in human pleural mesothelial cells associated with *Mycobacterium tuberculosis* infection and that this process might be implicated in the pathogenesis of tuberculous pleuritis [22]. TNF-α is a strong activator for NF-κB, and MMP-9 expression is known to be upregulated via activation of NF-κB signaling in various types of cells [23,24]. The signaling pathway involved in the enhanced MMP-9 expression in mesothelial cells should be clarified in future studies.

The origin of TNF-α involved in the stimulation of mesothelial cells is unclear at present. Considering that micrometastasis in the early stage of peritoneal dissemination takes place mainly in the omentum, where many small lymphoid tissues called milky spots are present [25,26], macrophages and lymphoid cells accumulated in milky spots are likely to secrete TNF-α. Another candidate would be the macrophages infiltrating into tumors, which are collectively called tumor-associated macrophages (TAMs) and are known to produce various cytokines depending on the stage and types of tumors [16,27,28].

A recent clinical study on patients with gastric cancer indicated a positive correlation between TNF-α expression and peritoneal metastasis [14]. The results showed that the TNF-α expressions were significantly different between the metastatic group and non-metastatic group, and the logistic regression analysis indicated that TNF-α expression was a risk factor for peritoneal metastasis of gastric cancer. TNF-α has been shown to deteriorate malignant phenotypes of cancer cells, including cell proliferation, migration, epithelial-mesenchymal transition, and productivity of cytokines and growth factors. In addition to these effects on cancer cells, it seems important that the phenotypic changes of mesothelial cells induced by TNF-α, especially the enhancement of MMP-9 secretion, may cause peritoneal metastasis, and thus a poor prognosis for patients with gastric cancer. The relationship between the level of MMP-9 in the gastric carcinoma environment and the malignant invasive phenotypes of gastric carcinoma, including lymph node metastasis, has been extensively studied, and the data suggest that MMP-9 plays important roles in the progression of gastric cancer [29,30,31]. Therefore, it would be feasible that MMP-9 and its endogenous inhibitors (tissue inhibitors of metalloproteinases, TIMPs) are preventive and therapeutic targets for cancer metastasis.

We have previously studied the interaction of gastric carcinoma cells with the mesothelium and found that the integrin-dependent adhesion of carcinoma cells to the submesothelial matrix promotes MMP-9 secretion from cancer cells [15]. TNF-α treatment of mesothelial cells has been shown to induce morphological changes characterized by cell rounding and disruption of the cell–cell contacts, leading to the expansion of intercellular gaps [19]. It is likely that the adhesion of cancer cells to the exposed submesothelial matrix is stabilized, which might promote further upregulation of MMP-9 secretion and successive invasion of cancer cells. In conclusion, the present study strongly suggests that TNF-α-induced MMP-9 secretions from mesothelial cells play an important role in the metastatic dissemination of gastric cancer.

## 4. Materials and Methods

### 4.1. Reagents

RPMI1640 medium, trypsin, Triton X-100, Brij 35, TriReagent and hexadimethrine bromide were purchased from Sigma-Aldrich (St. Louis, MO, USA). Nonidet P-40 was from Nacalai Tesque (Kyoto, Japan). Bovine serum albumin (BSA), gelatin, dimethyl sulfoxide (DMSO), phorbol 12-myristate 13-acetate (PMA) and polyethylene glycol (PEG) 6000 were purchased from Wako Pure Chemical Industries (Osaka, Japan). Polyethylenimine “Max” was purchased from Polysciences Inc. (Warrington, PA, USA). Fetal calf serum (FCS) was obtained from Biosera (Boussens, France). ASF104 serum-free medium was supplied by Ajinomoto (Tokyo, Japan). Matrigel was purchased from BD Biosciences (San Diego, CA, USA). The PrimeScript RT Reagent Kit and KAPA SYBR FAST qPCR Kit Master Mix (2×) ABI Prism were obtained from Takara Bio Inc. (Shiga, Japan) and KAPA Biosystems (Boston, MA, USA), respectively. ViraPower Lentiviral Packaging Mix and puromycin dihydrochloride were supplied by Life Technologies (Carlsbad, CA, USA). Oligonucleotides were supplied by FASMAC (Kanagawa, Japan). Recombinant murine TNF-α was a product of Peprotech (Rocky Hill, NJ, USA). A fluorescent dye, 3′-*O*-acetyl-2′, 7′-*bis*(carboxyethyl)-4 or 5-carboxyfluorescein, diacetoxymethyl ester (BCECF-AM), was obtained from Dojindo Laboratories (Kumamoto, Japan). Gelatin Sepharose 4B was a product of GE Healthcare (Piscataway, NJ, USA).

### 4.2. Cells

The MKN1 human gastric carcinoma, HT1080 human fibrosarcoma, and THP-1 human acute monocytic leukemia cell lines were supplied by the RIKEN Cell Bank (Tsukuba, Japan) and cultured in RPMI 1640 medium supplemented with 10% FCS at 37 °C under 5% CO_2_. Mesothelial cells were isolated under sterile conditions essentially as described by Nakashio et al. [32]. Briefly, the parietal peritoneum and diaphragm excised from ddY mice were washed with phosphate-buffered saline (PBS) and incubated with 0.25% trypsin in PBS at 37 °C for 30 min with gentle mixing every 5 min. After an equal volume of RPMI 1640/10% FCS was added to the cell suspension, the mixture was centrifuged at 2000 rpm for 10 min. The pelleted cells were suspended and incubated with ammonium-chloride-potassium (ACK) lysis buffer (150 mM NH_4_Cl, 10 mM KHCO_3_, 0.1 mM EDTA, pH 7.4) to remove red blood cells at room temperature for 1 min. After 10 volumes of RPMI 1640 medium/10% FCS was added, the mesothelial cells were recovered by centrifugation at 2000 rpm for 10 min. The pelleted cells were resuspended in RPMI 1640 medium/10% FCS and passed through nylon mesh (40 μm), and an aliquot (2 × 10^5^ cells/0.1 mL) was placed in a 96-well culture plate that had been coated with 10% Matrigel at 4 °C for 16 h. The cells were cultured at 37 °C for 4–5 days until the growth of a monolayer of polygonal mesothelial cells. This procedure was conducted in accordance with the Guide for the Care and Use of Laboratory Animals of Hoshi University School of Pharmacy and Pharmaceutical Sciences (accredited by the Ministry of Education, Culture, Sports, Science and Technology, Japan).

### 4.3. Gelatin Zymography

MMP activity was assayed by gelatin zymography essentially as described previously [33]. Briefly, MKN1 and/or mesothelial cells were treated with TNF-α (0, 1, 10, 100 ng/mL) in ASF104 serum-free medium for 0–48 h, and the culture supernatants were mixed with sample buffer for SDS-polyacrylamide gel electrophoresis (Laemmli’s buffer without reducing agent). Specimens were electrophoresed on a polyacrylamide gel (7.5%) containing 0.15% gelatin. The gel was washed three times with 1% Triton X-100 for 20 min each, and three times with water for 20 min each. Then gelatinolytic activity was detected by incubating the gel for 16 h at 37 °C in 50 mM Tris-HCl, 150 mM NaCl, 5 mM CaCl_2_, and 1 μM ZnCl_2_ followed by staining with Coomassie brilliant blue (CBB) R-250 (Merck, Darmstadt, Germany).

### 4.4. Quantitative RT-PCR (RT-qPCR)

Total RNA was isolated from mesothelial cells using TriReagent, and cDNA was synthesized with a PrimeScript RT reagent kit using the total RNA as a template according to the manufacturer’s protocols. The qPCR reactions were conducted with an Applied Biosystems StepOne system (Life Technologies) using a KAPA SYBR FAST qPCR Kit Master Mix (2×) ABI Prism under the following conditions: Denaturation at 95 °C for 20 s followed by 40 cycles of 95 °C for 5 s, 55 °C for 30 s and 72 °C for 30 s. Melting curve analysis and agarose gel electrophoresis of the PCR products were performed to verify the specific amplification. All samples were analyzed in triplicate and quantified by the relative standard curve method using the *Gapdh* housekeeping gene as an internal control. The sequences of the primers used were as follows: *Mmp2*, 5′-ATC GCT CAG ATC CGT GGT G-3′ (forward) and 5′-GGA GCT CAG GCC AGA ATG T-3′ (reverse); *Mmp9*, 5′-ATG TAC CCG CTG TAT AGC TAC C-3′ (forward) and 5′-ATA GTG GGA CAC ATA GTG GGA G-3′ (reverse); *Gapdh*, 5′-TGA AGC AGG CAT CTG AGG G-3′ (forward) and 5′-CGA AGG TGG AAG AGT GGG AG-3′ (reverse).

### 4.5. Cell Invasion Assay Using Reconstituted Mesothelium

Reconstituted artificial mesothelium consisting of a monolayer of mesothelial cells on a Matrigel layer in a Boyden chamber (Transwell) system was utilized for cell invasion assay. Murine mesothelial cells were isolated as described above and cultured in a 10% Matrigel-coated inner chamber with a polyethylene terephthalate (PET) membrane (pore size, 8 μm; Falcon 353097; BD Biosciences) combined with an outer chamber (Falcon 353504) for 4–5 days until the cells grew as a confluent monolayer. An MKN1 gastric carcinoma cell suspension (1 × 10^5^ cells/0.2 mL) was placed in the inner (upper) chamber, and the outer (lower) chamber was filled with ASF104 serum-free medium supplemented with HT1080 serum-free conditioned medium. The addition of HT1080 conditioned medium (20%) increased the chemotactic activity, although the conditioned medium contained a moderate level of MMP-9 secreted by HT1080 cells. After the chambers were incubated at 37 °C for 16 h, the cells that had migrated into the lower chamber through the membrane were stained with Diff-Quik (International Reagents, Kobe, Japan) and counted under a microscope.

### 4.6. Cell Adhesion Assay

MKN1 cells were labeled with the fluorescent dye BCECF-AM (3 μM) at 37 °C for 40 min [18]. The labeled cells (1 × 10^5^ cells/0.1 mL in 1% BSA containing RPMI 1640 medium) were added to the monolayer of mesothelial cells in a 96-well culture plate as described above. After the plate was incubated at 37 °C for 40 min, non-adherent cells were removed by gentle washing three times with PBS. Adherent cells were lysed with 0.1 mL of 1% Nonidet P-40, and the lysates were diluted with 9 volumes of PBS. The fluorescence intensities were measured with a fluorescence spectrophotometer (Ex = 490 nm, Em = 520 nm). The percent adhesion was calculated as follows: (The fluorescence intensity of adherent cells/fluorescence intensity of total cells) × 100.

### 4.7. Flow Cytometry

The expression of α3 integrin in MKN1 cells was measured by a flow cytometer (BD FACSVerse; BD Biosciences, San Diego, CA, USA), using monoclonal antibodies against human integrin α3 subunit (SM-T1) [34] and FITC-labeled anti-mouse IgG (Kirkegaard & Perry Laboratories, Gaithersburg, MD, USA).

### 4.8. MMP-Knockdown MKN1 Cells

Knockdown of MMP-2 and MMP-9 was conducted by using lentivirus-mediated RNA interference. The short hairpin RNAs (shRNAs) of target sequences against MMP-2 (185–203: AGG AGA GCT GCA TCC TGT T) [35] and MMP-9 (343–361: AAG TGG TAC CAC CAT AAC A) [36] were cloned into the pSIH-H1 vector (System Biosciences, Palo Alto, CA, USA) according to the manufacturer’s protocol. Lentiviral particles were produced in HEK293FT cells after the co-transfection of pSIH-H1 vector and ViraPower (three packaging plasmids) using polyethylenimine “MAX” [37] for 48 h and concentrated by the PEG precipitation method. Briefly, the culture medium containing viral particles was mixed with a one-third volume of 4 × PEG solution (32% PEG, 400 mM NaCl, 40 mM HEPES-NaOH, pH 7.4) and incubated at 4 °C overnight. After centrifugation (3000× *g*, 30 min), the obtained precipitate was dissolved in serum-free RPMI 1640 medium. The virus solution was mixed with hexadimethrine bromide (8 μg/mL at final concentration), and the mixture was added to pre-cultured MKN1 cells (1 × 10^5^ cells/2 mL in a 6-well plate). The plate was then centrifuged (1800× *g*, 90 min) at 32 °C and incubated at 37 °C for 48 h. The cells were successively cultured for 24 h after the medium was replaced with RPMI 1640 medium/10% FCS, followed by selection with puromycin (2.5 μg/mL).

### 4.9. Purification of MMP-9

MMP-9 was purified from the conditioned medium of human monocytic leukemia THP-1 cells based on the method of Morodomi et al. [38]. THP-1 cells were cultured in ASF104 medium containing PMA (5 nM) at 37 °C for 3–4 days. The conditioned medium was mixed with gelatin Sepharose 4B which had been equilibrated with TNC buffer (50 mM Tris-HCl, 0.15 M NaCl, 10 mM CaCl_2_, 0.05% Brij 35, pH 7.5) and incubated at 4 °C for 16 h with gentle agitation. After gelatin Sepharose 4B was washed three times with TNC buffer, MMP-9 was eluted with DMSO (5%)-containing TNC buffer. The eluate was then applied to a PD-10 desalting column (GE Healthcare, Piscataway, NJ, USA) to replace the buffer with ASF104 medium. The purity of MMP-9 was estimated to be >95%, as analyzed by SDS-polyacrylamide gel electrophoresis followed by CBB and silver staining. The amino acid sequence of the purified protein was analyzed by peptide mass fingerprinting. The tryptic fragments obtained from the specimen were applied to MALDI/TOFMS (AXIMA-QIT; Shimadzu/Kratos, Kyoto, Japan) as described previously [39]. Major mass peaks detected by the MS analysis and the corresponding amino acid sequences to human MMP-9 were as follows: *m*/*z* 1345.74 for ^25^QSTLVLFPGDLR^36^, *m*/*z* 1184.62 for ^43^QLAEEYLYR^51^, *m*/*z* 1701.90 for ^77^QLSLPETGELDSATLK^92^, *m*/*z* 1084.55 for ^107^FQTFEGDLK^115^, *m*/*z* 1680.91 for ^144^AFALWSAVTPLTFTR^158^, *m*/*z* 2350.10 for ^163^DADIVIQFGVAEHGDGYPFDGK^184^, *m*/*z* 3175.37 for ^185^DGLLAHAFPPGPGIQGDAHFDDDELWSLGK^214^, *m*/*z* 2012.89 for ^222^FGNADGAACHFPFIFEGR^239^, *m*/*z* 2073.87 for ^250^SDGLPWCSTTANYDTDDR^267^, *m*/*z* 999.47 for ^268^FGFCPSER^275^, *m*/*z* 997.50 for ^325^ LFGFCPTR^332^, *m*/*z* 1385.79 for ^547^GSRPQGPFLIADK^559^, *m*/*z* 739.30 for ^560^WPALPR^565^, *m*/*z* 873.45 for ^579^LFFFSGR^585^, *m*/*z* 1532.83 for ^586^QVWVYTGASVLGPR^599^, *m*/*z* 1440.80 for ^604^LGLGADVAQVTGALR^618^, *m*/*z* 823.45 for ^624^MLLFSGR^630^ and *m*/*z* 1921.91 for ^653^MFPGVPLDTHDVFQYR^668^ (UniProt P14780). Based on these results, the purified protein was confirmed to be MMP-9.

## Supplementary Materials

Supplementary materials can be found at http://www.mdpi.com/1422-0067/19/12/3961/s1. Figure S1: RT-qPCR analysis of mRNA for the γ2 subunit of laminin-332 in mesothelial cells; Figure S2: Purification of MMP-9 from THP-1 cells; Figure S3: Zymographic analysis of MMPs in the conditioned medium of MMP-2- or MMP-9-knockdown MKN1 cells.

## Abbreviations

BCECF-AM	3′-*O*-acetyl-2′,7′-*bis*(carboxyethyl)-4 or 5-carboxyfluorescein, diacetoxymethyl ester
CBB	Coomassie brilliant blue
ECM	extracellular matrix
FCS	fetal calf serum
MALDI/TOFMS	matrix-assisted laser desorption ionization/time-of-flight mass spectrometer
MMP	matrix metalloproteinase
PBS	phosphate-buffered saline
PMA	phorbol 12-myristate 13-acetate
RT-PCR	reverse transcription-polymerase chain reaction
TNF-α	tumor necrosis factor-α
