# Supplementary material for: Stimulation of Peritoneal Mesothelial Cells to Secrete Matrix Metalloproteinase-9 (MMP-9) by TNF-α: A Role in the Invasion of Gastric Carcinoma Cells
Source: Int J Mol Sci. 2018 Dec 9;19(12):3961. doi: 10.3390/ijms19123961 (PMC6321609; doi:10.3390/ijms19123961)
Supplement: Supplementary file 1 [file ijms-19-03961-s001.pdf]

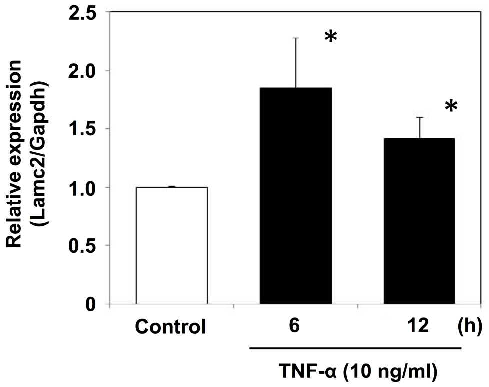

Figure S1. RT-qPCR analysis of mRNA for the $\gamma 2$ subunit of laminin-332 in mesothelial cells after the treatment with TNF-α (10 ng/ml) for 6 or 12 h. Experiments were performed in triplicate, and the data are presented as the mean ± SEM. Statistical data analysis was conducted using the Student's t-test. *$p < 0.05$.

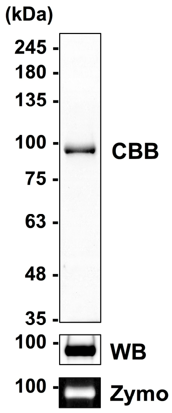

Figure S2. Purification of MMP-9 from THP-1 cells. An MMP-9 specimen purified from the conditioned medium of THP-1 cells was analyzed by SDS-PAGE/CBB staining, western blotting with anti-MMP-9 antibody, and zymography.

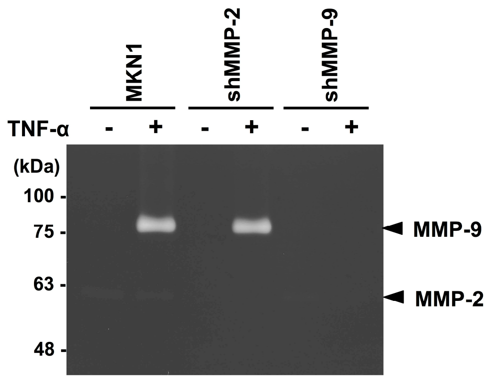

Figure S3. Zymographic analysis of MMPs in the conditioned medium of MMP-2-knockdown (*shMMP-2*) or MMP-9-knockdown (*shMMP-9*) MKN1 cells.
